# Evaluation of an enrichment programme for a colony of long-tailed macaques (*Macaca fascicularis*) in a rescue centre

**DOI:** 10.1007/s10329-021-00908-8

**Published:** 2021-04-11

**Authors:** Valeria Albanese, Michela Kuan, Pier Attilio Accorsi, Roberta Berardi, Giovanna Marliani

**Affiliations:** 1 LAV, Viale Regina Margherita, 177, 00198 Roma, Italy; 2 Dipartimento di Scienze Mediche Veterinarie, Università di Bologna, Via Tolara di Sopra 50, 40064 Ozzano Emilia, BO Italy

**Keywords:** *Macaca fascicularis*, Enrichment, Cortisol, Behaviour, Welfare

## Abstract

Long-tailed macaques are highly social primates that are commonly used in biomedical research as animal models. The aim of this study was to evaluate the effects of different kinds of enrichment on the behaviour and faecal cortisol metabolite (FCM) level in a colony of ex-laboratory long-tailed macaques during a programme of rehabilitation. The research was carried out in three periods, divided into two sessions each. Every period was composed of one control session (SC) and one session characterised by one type of enrichment: feeding enrichment (FE), manipulative enrichment (ME), and the last session during which manipulative and feeding enrichment were provided every day but in a mixed way (MIX). The results showed that manipulative and mixed enrichments caused positive changes to the activity budget of the colony, with a decrease in abnormal behaviour rates and an increase in play compared with control sessions. The rate of affiliative behaviours and low rate of aggression were probably because the group was composed mostly of females and it was stable, with a well-defined hierarchy. The research underlines the importance of a well-studied enrichment programme for the welfare of captive animals, which should exploit species-specific motivations.

## Introduction

Non-human primates are still widely used for experimental purposes in Italy (GU no. 28 of 02-02-2019), despite strong constraints on their use by national and international rules and evidence of important currents of ethical opposition to their use at the global level. Indeed, the European Commission has produced a very restrictive report on the subject (Adams et al. 2019), while an independent Dutch institute has even stated that the use of monkeys should be halted immediately, calling it an unsustainable model, not only because of ethical issues, but also for scientific and legal reasons (Koëter et al. 2016).

Although European Directive 63 of 2010 provides for the release and rehoming of animals, the number of animals recovered at the end of procedures is very low and usually involves species such as mice, rats, rabbits, gerbils and dogs, which can be introduced into a family and adopted individually (Bucchino 2020). This cannot happen with non-human primates—exotic species that need a special environment and the keeping of which at home is forbidden in most European countries (Law n. 150/1992; Reg. CE n. 338/1997 e n. 101/2012). The lab’s housing conditions, to which animals can be subjected for many years, can cause physical and behavioural changes (e.g. alopecia and abnormal behaviours) in primates (Lutz 2018; Kroeker et al. 2014). The presence of abnormal behaviours can make the rehabilitation of the animals difficult (Cheyne 2006). However, enrichment can be successfully used to encourage natural species-specific behavioural repertoires, to decrease the rate of abnormal behaviour and to improve welfare in animals under human care (Lutz and Novak 2005; Gronqvist et al. 2013). To be effective, a good enrichment programme should consider the species-specific natural history and ecological features of the animals (Lutz and Novak 2005). For example, in primates such as macaques, social housing can be considered a good enrichment itself, and in stressful situations, the presence of companions can work as a buffer (Hennessy et al. 2016; Hannibal et al. 2017). Indeed, long-tailed macaques (*Macaca fascicularis*), widely distributed in Southeast Asia, live in multi-male/multi-female groups, which can include up to 40 individuals (van Noordwijk and van Schaik 1985). Other examples of enrichment can be structural enrichment (such as platforms and water pools), feeding enrichment (i.e. new food items or puzzle feeders) and manipulative enrichment (toys and novel objects) (Cannon et al. 2016). These kinds of enrichment can be useful when it is not possible to keep animals in groups or to lower the rate of abnormal behaviour and in cases of aggression (Cannon et al. 2016; Wooddell et al. 2019). The evaluation of stressful situations should consider not only behavioural parameters, but also physiological indices (Rushen et al. 2011), such as cortisol concentration. Indeed, stressful conditions cause the activation of the hypothalamic–pituitary–adrenal (HPA) axis and the release of cortisol (Moberg 2000). Therefore, the measurement of cortisol and its metabolites in faeces is widely employed in animal welfare studies, as useful non-invasive method to assess the level of stress of animals (Palme 2005, 2012; Schwarzenberger 2007).

Rescue centres, thanks to the support of primatologists and veterinarians, accept animals that come from labs and try to work to achieve physical and behavioural rehabilitation (Carlsson et al. 2004). Rehabilitation programmes should understand and consider the species-specific physiological, psychological, and behavioural needs of the animals and guarantee a state of welfare for the subjects that will be maintained in rescue centres. In Italy, two pilot rehabilitation programmes consisting of two colonies of long-tailed macaques that came from laboratories are currently active, in an effort to create a model that can be applied in the recovery of primates that came from labs. The aim of this study was to evaluate the effect of different kinds of enrichment on the behaviour and welfare of the animals belonging to one of the Italian colonies, in order to improve rehabilitation programmes for rescued macaques. To have a multidisciplinary approach, it was decided to combine observational data with the evaluation of the faecal cortisol metabolite (FCM) level of the colony, two non-invasive methods for monitoring the welfare of animals.

## Methods

### Study sites and subjects

The subjects of the study are part of a group of 27 macaques that came from an Italian laboratory in July 2017. They came from non-EU breeding facilities (China, Philippines and Mauritius) and were kept in the laboratory for 10 years without undergoing experimentation before being released to the rescue centre. In the laboratory facilities, animals were housed in internal cages without an external enclosure. Males were single-caged and they were separated from the females, which were kept in two different groups. Animals were fed a pellet-based diet and they were maintained according to the standard requirements established by Annex III of Decree 26/2014 (National Competent Authorities for the implementation of Directive 2010/63/EU).

The observations for the study started one and a half years after the macaques’ arrival at the rescue centre. Before the beginning of the study, one of the males and three females died due to health issues. In addition, another male, suffering from diabetes, was placed in a separate enclosure with another female. The study group was therefore composed of one sterilised adult male and 20 adult female long-tailed macaques (no. 21), aged from 9 to 22 years (12 ± 3 years). On 26th July 2019, the alpha female died from natural causes, so during the second period of the study the subjects numbered 20. All animals of the group considered were housed together in the same enclosure. They were fed four times a day with pellet food (Kasper Faunafood), distributed in the morning, and fruits, mixed vegetables and carrots during the rest of the day. Food was distributed both in the feeders and thrown from above the mesh. Their daily diet was the same throughout the study. The enclosure of the rescue centre consists of two inside sections (36 m^2^) connected by a hatch placed on the ground; each of the enclosures connects with the outside through another three hatches. The animals can see outside through two windows placed on each side. The inside enclosures include different wooden platforms and hammocks made from fire hoses. The ground is covered with sawdust (10 cm deep) and there are four drinking sites. There is one main outside enclosure (450 m^2^) furnished with wooden trunks, wooden walls and a pool (20 cm deep), and a second outside enclosure that measures 30 m^2^. In the outside enclosures, the ground is of natural soil, and the fences are made of plasticised metal mesh. The macaques always have access to the outside.

### Enrichment

In this research we considered three periods in which a controlled and standardised enrichment programme was set up, which was made by alternating control sessions (SC) and sessions with different kinds of enrichment. One type of enrichment was provided at a time, but it was different for each day.

All the items used during the enrichment sessions of the study were totally new to the macaques.

The first session of enrichment was characterised by feeding enrichment (FE), which included only food that was not part of their usual diet (e.g. seeds, rice, frozen fruit, cooked cornflour, pine cones), scattered on the ground or placed directly on the structures of the enclosures.

During the second session with manipulative enrichment (ME), only objects that they could inspect or interact with were used (e.g. plastic tubes, textiles, stones, interactive wooden boards, metal items such as bowls and spoons, that they could lick or use to look at themselves). Since they are naturally used to handling objects, they would also play with them, by piling them up or rubbing them on the ground.

**Fig. 1:**
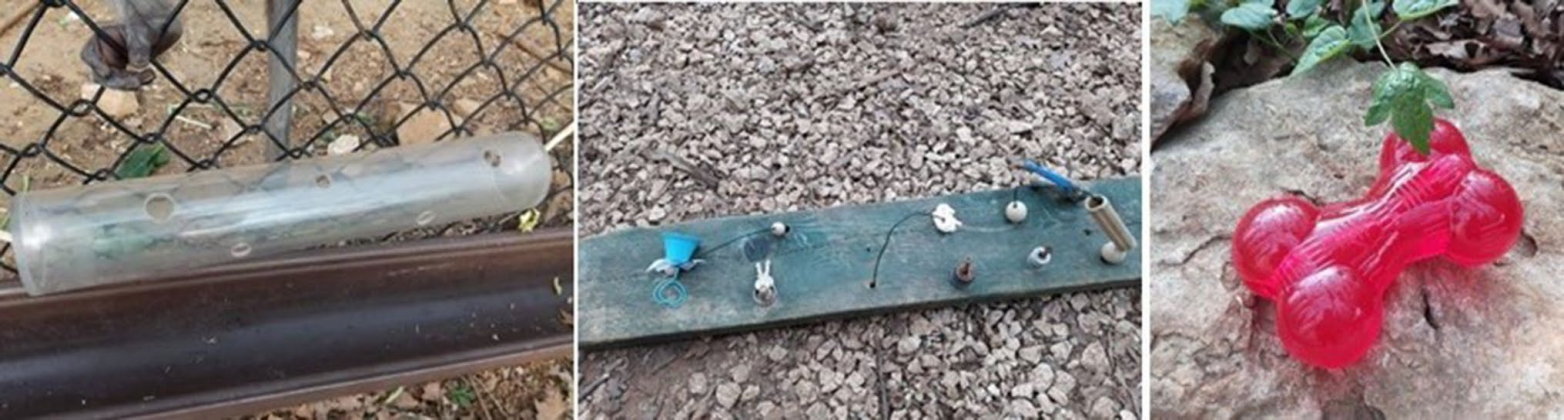
Examples of feeding devices and manipulative enrichments: feeders used in MIX session, interactive wooden board used in ME session and Kong filled with food used in MIX session

Finally, in the third session of enrichment there was mixed enrichment (MIX), consisting of different types of enrichment, i.e. feeding devices (e.g. Kongs, plexiglass tubes with food inside), unusual foods scattered on the ground and manipulative enrichment (Fig. 1).

### Data collection

The observations took place between December 2018 and December 2019, and comprised three periods, each divided into two sessions. Each session was characterised by the absence or the presence of a new enrichment type (Table 1).

**Table 1:** Periods, sessions and number of subjects considered in the research

Period	Session	Number of days per session	Number of subjects
1 6th December 2018–10th January 2019	SC 1	10	21
	FE	11	21
2 14 January 2019–4th March 2019	SC 2	12	21
	ME	12	21
3 5th November 2019–29th November 2019	SC 3	14	20
	MIX	12	20

*SC* control session, the session of control without enrichment, *FE* session with feeding enrichment, *ME* session with manipulative enrichment, *MIX* session with mixed enrichment

Focal animal sampling was employed, and during each session, which lasted from 9 to 14 days, each animal was observed for 30 min, for a total of 4320 min. Every day the order of focal subjects was random. Between control sessions and the respective enrichment sessions, no more than 9 days passed. Observations during control sessions were always made between 9.55 a.m. and 3.50 p.m. During the enrichment session, items were distributed between 10:30 a.m. and 11:30 a.m. and observations were made always at least on an hour and a half after enrichment distribution. The data were recorded by the observer present in person on a on a sheet of paper, without video recording. A continuous recording method was used such that each behaviour from the ethogram (Table 2) was a state, and its duration and frequency were recorded considering the change in behaviour. After the observation, all data recorded were transcribed into Excel.

**Table 2:** Ethogram employed in the study (Nickelson and Lockard 1978; Pomerantz et al. 2012; Xu et al. 2012; Sha and Hanya 2013)

Ingestion behavior	Eating, looking for food and its manipulation. Foraging, feeding and drinking
Affiliative behavior	Friendly actions between individuals, e.g. allogrooming (receiving grooming from or carrying out grooming on another subject and grooming each other)
Reproductive behavior	Pelvic mounting observed during heterosexual or homosexual encounters and sexual interactions
Aggressive behavior	Physically attacking another subject (e.g. biting), chasing or threatening
Vocalisation	Vocal sound produced as a means of communication in agonistic or affiliative context or as alert behaviour
Self-grooming	Maintenance behaviour. The animal licks, nips and rubs itself to groom itself
Abnormal behavior	Anomalous behaviour including stereotypic pacing and biting the mesh
Play	Amicable behaviour directed towards another individual that includes play face, play chasing, play biting and non-social play, e.g. object manipulation
Locomotion	Moving to another location by walking, scampering, scurrying or climbing
Rest	The animal is inactive, sleeping, laying down, or sitting, while looking around
Miscellaneous behaviour (other)	Behaviours which are unclear in their meaning or observed in multiple contexts or did not fit in any of the other categories

### Faecal sampling and faecal cortisol metabolite assays

Faecal samples were collected from the inside enclosure during the morning and a total of 16 samples were analysed (SC1 = no. 4; FE = no. 4; SC3 = no. 4; MIX = no. 4). The samples were collected between December 2018 and January 2019 and between November and December 2019, and they were collected at least 2 days after the beginning of the respective sessions of observation and spread throughout the session. During SC2 and ME, it was not possible to collect enough samples to consider in the analysis. Each sample, which represented a pooled sample of the entire colony, was stored in a freezer (−18 °C) until delivery to the laboratory at the Department of Medical Veterinary Sciences of the University of Bologna (−20 °C). The mean and standard error of FCM concentration was calculated for each session, and because of the low number of samples, a statistical comparison was not performed.

Faecal cortisol metabolite levels were determined by radioimmunoassays (RIAs). The extraction methodology was modified from Schatz and Palme (2001). Cortisol metabolite assays in faeces were carried out according to Tamanini et al. (1983). Validation parameters of analyses were as follows: sensitivity 0.19 pg/mg, intra-assay variability 5.9%, inter-assay variability 8.7%. Radioactivity was determined using a liquid scintillation beta counter and a linear standard curve, designed ad hoc by a software programme (Motta and Degli Esposti 1981). All concentrations were expressed in picograms per milligram of faecal matter.

### Data analyses

All data were transferred to Excel sheets and analysed, calculating the mean and the standard error (SE) of frequency and percentage duration of each behaviour, and the mean ± SE of FCM concentrations in each session.

A normality test (Shapiro–Wilk test) was performed to verify the distribution of frequency and duration data. Because the data were not normally distributed, the Wilcoxon pairwise test was used to statistically compare the frequency and the duration of each behaviour between the two sessions of the three different periods. Indeed, the aim of the research was to assess the potential efficacy of each enrichment.

All statistical analyses, the calculation of median and interquartile range (IQR), and the graphs were computed using the R version 4.0.2 software and an Excel program.

*p* values < 0.05 were considered statistically significant.

## Results

**Fig. 2:**
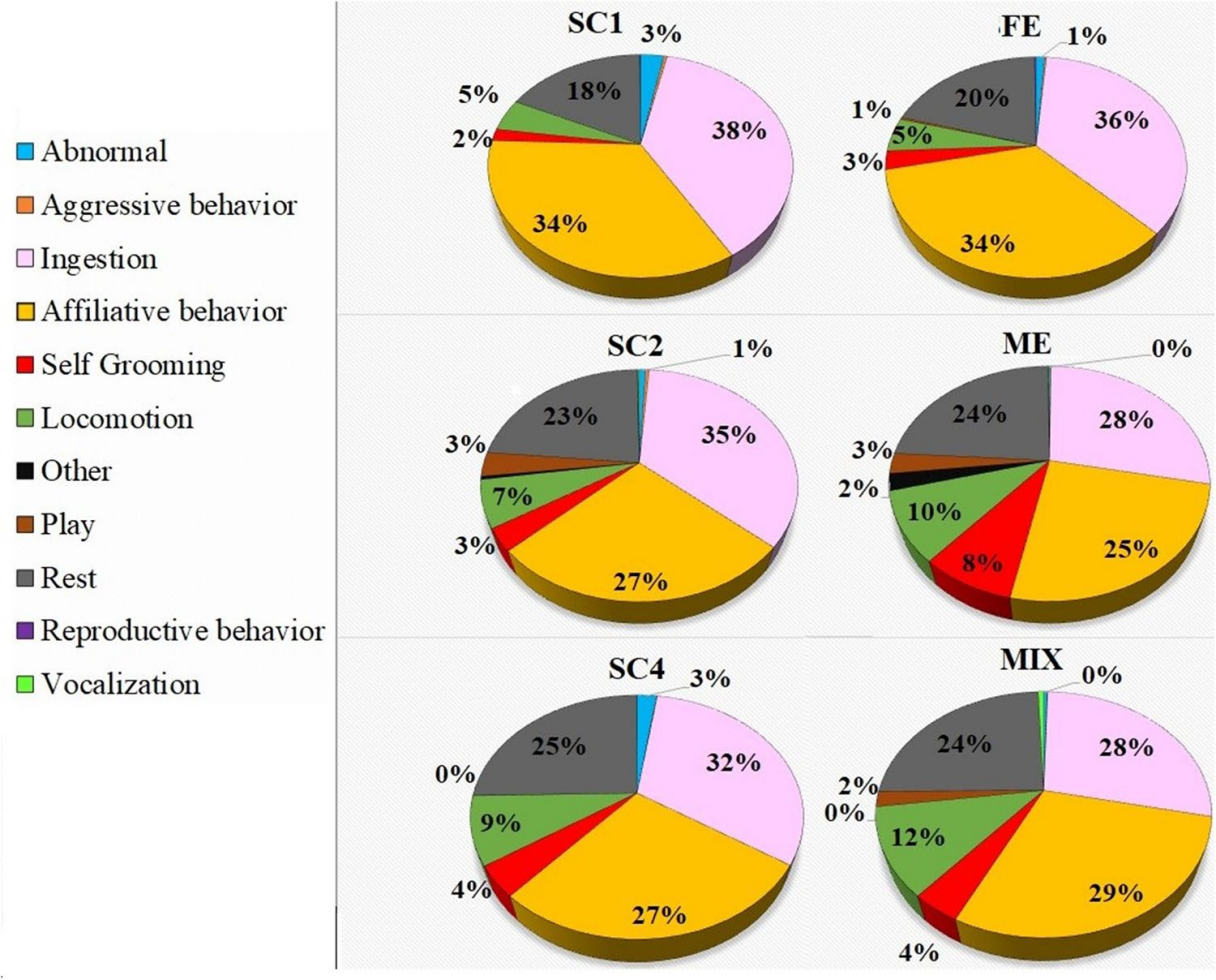
Activity budget of macaques during the three periods

During the three periods, six sessions of observations were conducted. Three control sessions, during which animals followed their daily routine, were alternated by three enrichment sessions: one during which the macaques had feeding enrichment (FE), one where the animals were provided with manipulative enrichment (ME), and the last during which both of the previous kinds of enrichment were employed (MIX). Each enrichment session was statistically compared with the control session that preceded it. The percentage activity budgets were calculated and are represented in the graphs of Fig. 2.

As shown in Fig. 2, during all the sessions the animals spent most of the time in ingestion or affiliative behaviour and resting. These behaviours did not change significantly during enrichment sessions compared to control sessions.

**Fig. 3:**
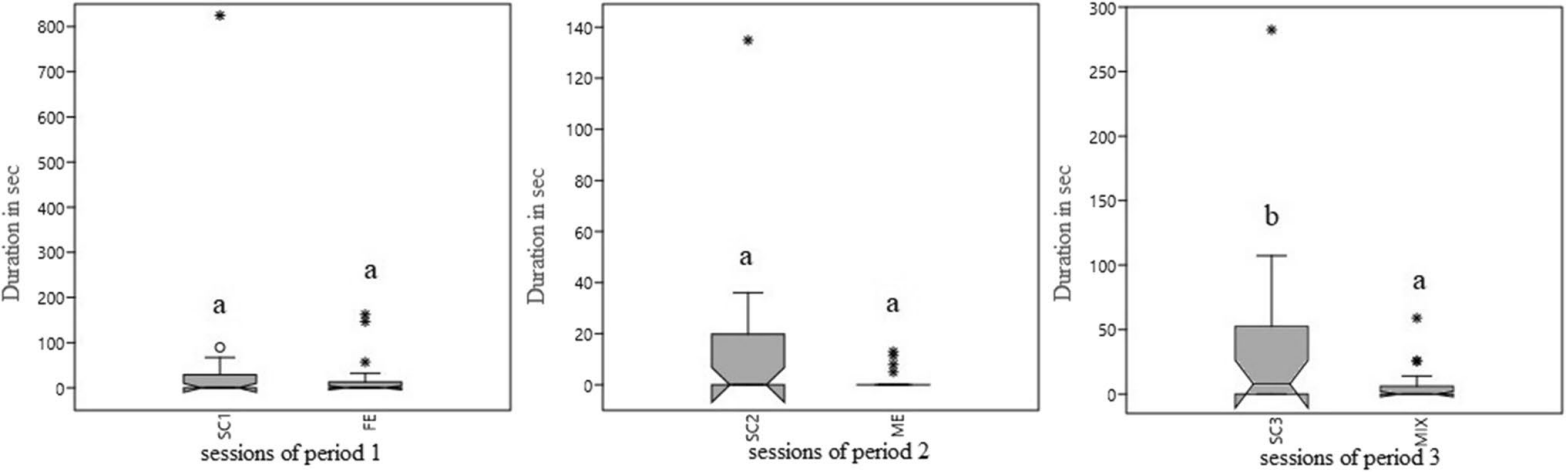
Boxplot of duration (in seconds) of abnormal behaviour during the three periods. **a**, **b** = *p* < .05. * = extreme outliers, ° = mild outliers

**Fig. 4:**
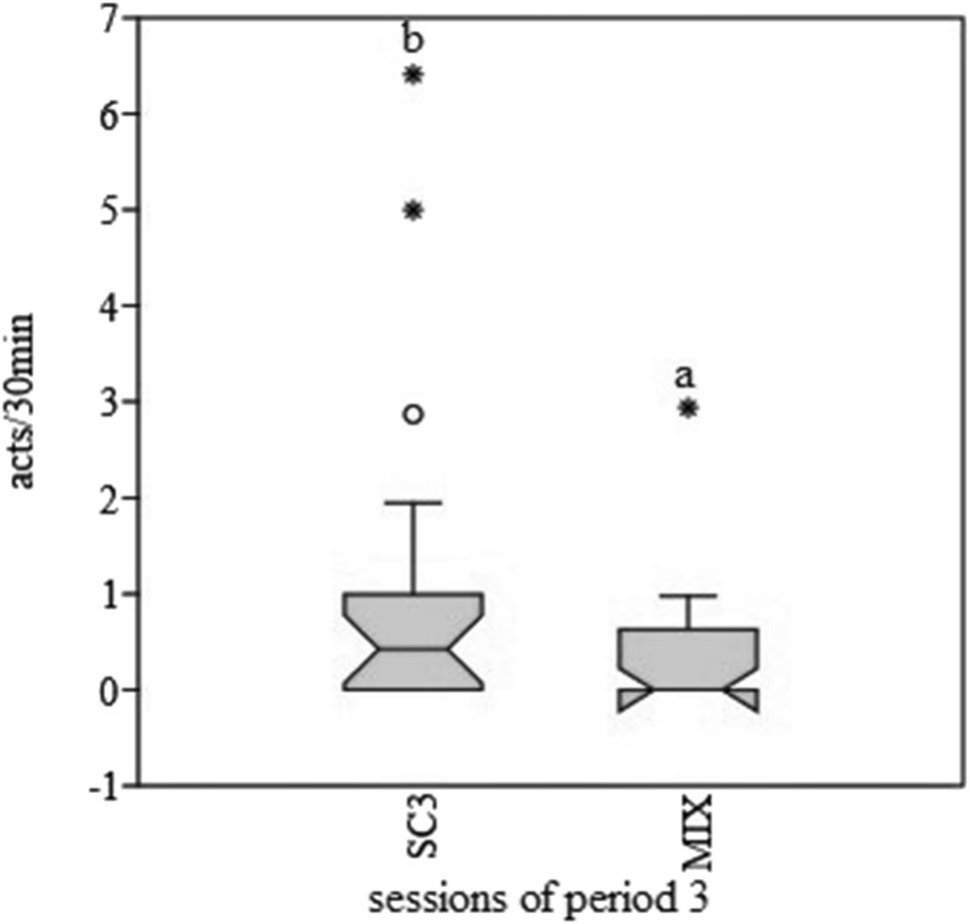
Boxplot of frequency (acts/30 min) of abnormal behaviour during SC3 and MIX. **a**, **b** = *p* < .05. * = extreme outliers, ° = mild outliers

The introduction of feeding enrichment did not significantly change the activity budget of macaques compared to SC1. During ME, there was a decrease, which approached significance, in the duration of abnormal behaviour (*p* = 0.07, W = 38, *Z* = 1.83) compared to SC2. The introduction of mixed enrichment caused a significant decrease of duration and frequency of abnormal behaviours (*p* = 0.03, W = 87, *Z* = 2.16; *p* = 0.02, W = 91, *Z* = 2.42) in comparison to SC3 (SC3 35.42 ± 14.71 s, MIX 6.59 ± 3.31 s; SC3 1.10 ± 0.40 acts/30 min, MIX = 0.33 ± 0.16 acts/30 min) (Figs. 3 and 4).

**Fig. 5:**
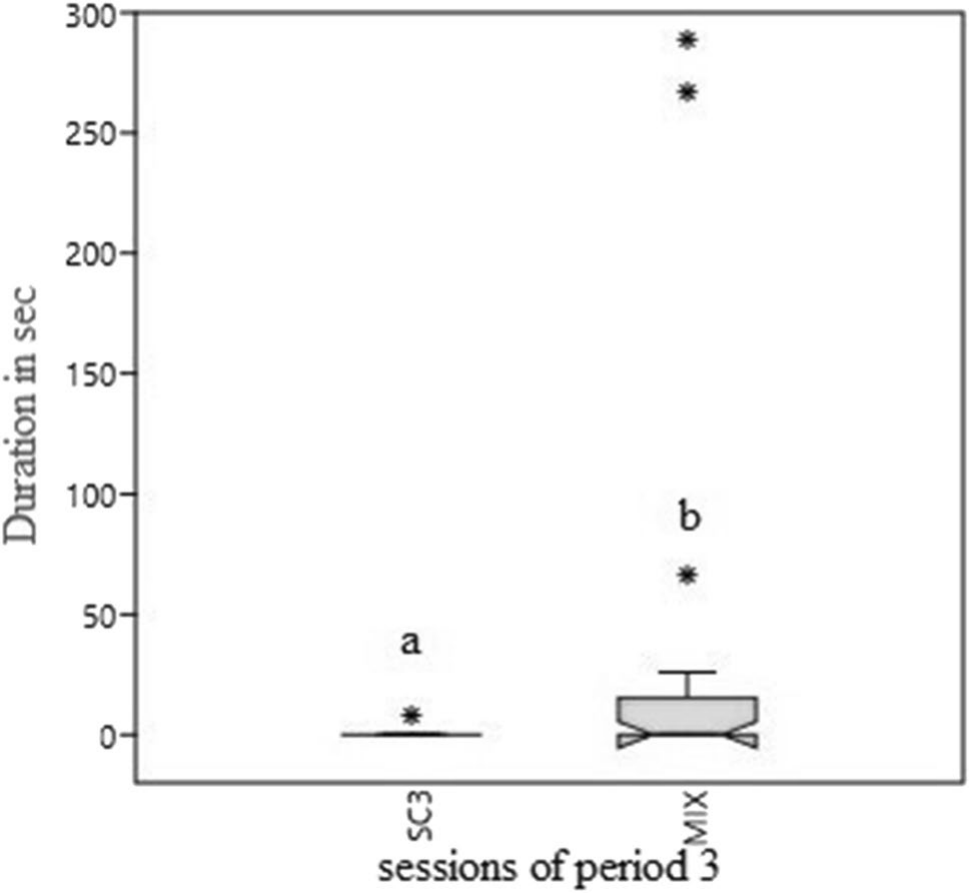
Boxplot of duration (seconds) of play behaviour in SC3 e MIX. **a**, **b** = *p* < .05. * = extreme outliers

Moreover, during MIX sessions an increase (*p* = 0.05, W = 26, *Z* = 2.03) was observed in the duration of play behaviour (SC3 0.40 ± 0.40 s, MIX 33.59 ± 19.03 s) (Fig. 5).

Aggressive behaviours were rare, and considering all the sessions, their frequency was less than 3 acts/min and they occupied less than 0.5% of the activity budget. Their frequency and duration did not change during enrichment sessions.

**Fig. 6:**
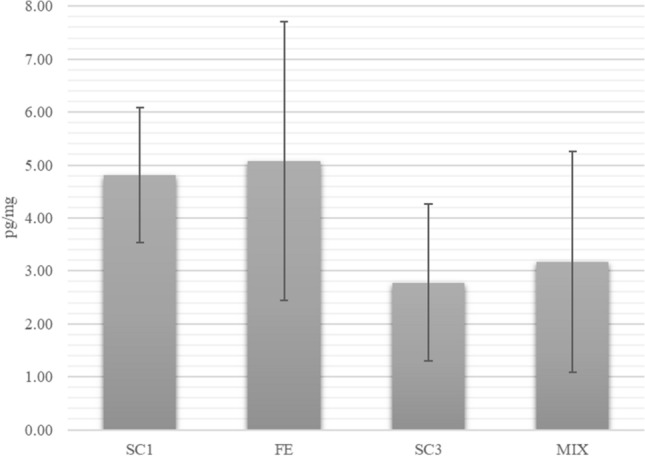
Mean and standard error of FCM concentration calculated in SC1, FE, SC3 and MIX

The results of FCM determination showed a slight increase in both FE and MIX sessions compared to the respective control sessions (SC1 4.81 ± 1.27 pg/mg, FE 5.08 ± 2.63 pg/mg; SC3 2.78 ± 1.48, MIX 3.17 ± 2.08 pg/mg) (Fig. 6).

## Discussion

Long-tailed macaques are Old World Monkeys, widely used as non-human primate models for biomedical research (Cauvin et al. 2015). Their inability to display species-specific behaviours can cause the development of a general stress condition, abnormal behaviour, depression, and aggressive behaviours (Mallapur et al. 2005). Therefore, it is important that centres that host ex-laboratory animals consider their ethological needs, to achieve success in their rehabilitation. The aim of the study was to consider the effectiveness of different kinds of enrichment included in a rehabilitation programme of macaques from labs. A good and well-studied enrichment programme can positively influence the welfare of captive animals and lower their stress levels, and it can decrease the rate of abnormal behaviours quite effectively (Honess and Marin 2006). In our research, the duration of abnormal behaviours in ME was lower than in SC2, with a significant tendency (*p* = 0.07), and during MIX, the frequency and duration of abnormal behaviour were significantly lower than in SC3. Abnormal behaviours, such as stereotypies and self-harm behaviours, are not present in the natural repertoire of the animals and can develop in captive conditions because the animal has no choice or control over its environment, cannot display its species-specific behaviours, and may therefore display such behaviours through boredom and frustration (Mason and Latham 2004; Balcombe et al. 2011; Novak 2013). These behaviours usually indicate poor welfare (Mason and Latham 2004). Considering all sessions of our study, the rate of abnormal behaviour did not exceed 3%, and it decreased during ME and MIX. These data can potentially support the idea of the reward effect due to the action of manipulating an object (Sambrook and Buchanan-Smith 1997; Jaman and Huffman 2008). Furthermore, it is suggested that the choice of enrichment should be made considering the behavioural needs and motivation of the species (Riley and Rose 2020).

Likewise, duration of play recorded in our study was higher (*p* = 0.05) in MIX than in SC3. Play is considered a possible indication of well-being in captivity (Held and Spinka 2011). This behaviour has no immediate goal and is energy-expensive. Animals in a captive situation display it when their needs are satisfied and there are no fitness threats, and it is also considered an expression of positive emotions (Held and Spinka 2011). Play in primates is often an expression of manipulative motivation and is itself a rewarding behaviour (Joey 1985; Held and Spinka 2011). Indeed, play is an opioid-mediated behaviour, and so it is a possible source of psychological pleasure. This behaviour gives psychological and health benefits, and in addition is socially contagious, so it can be a means for spreading good welfare (Held and Spinka 2011).

Social behaviours are fundamental behaviours in macaques, because of the gregarious and hierarchical nature of these animals (Thierry 2000). According to the results of this research, albeit in a sample of daily activity, affiliative behaviours (most of all allogrooming, but also other socially positive interactions) occupied an important percentage of the activity budget (25–34%). This supports the contention that macaques have a complex social system, with a high level of affiliative behaviours, also in captive conditions (Thierry 2000; Sussman et al. 2013). Moreover, females have demonstrated more contact proximity than males (Brent and Veira 2002) and a study by Crockett et al. in (1994) showed that pair-housed females spent more than one third of the day in social grooming, with psychological benefit for the animals. The high rate of affiliative behaviours and low rate of aggressions recorded in our study are probably due to the group being composed mostly of stable females, with a well-defined hierarchy (Bernstein et al. 1974).

In this study, feeding enrichment did not seem to evoke any significant behavioural change, probably because, even if new and unusual food items were given, the way they were distributed was like their usual feeding routine. However, when this kind of enrichment was alternated with manipulative enrichment or the food was put into devices (MIX), it had beneficial effects, such as the reduction of abnormal behaviours and an increase in play. One of the key characteristics of a good enrichment programme is novelty. Novelty can be both stressful and enriching at the same time, and it stimulates learning and exploring. However, when the animal finishes learning the properties of an object, the novelty ceases to exist and, with it, the enrichment effect (Buchanan-Smith 2010). The manipulative objects used as enrichment can easily lead to habituation and so they have a limited-time efficiency, but at the same time they give the animal the possibility to have control over its environment. To limit the problem of habituation, it is important to use different objects with different characteristics, to rotate the enrichment and to grant intermittent exposure to enrichment items (Honess and Marin 2006). All these characteristics were present during ME and MIX and the alternation with control periods could have contributed to the efficiency of the enrichment programme. The simultaneous presence of more than one object avoids the development of aggression (Honess and Marin 2006). In our research the presence of enough and non-concentrated food during FE and of more than one object during ME, meant that competition and an increase of aggressive behaviours were avoided.

Measurement of cortisol or its metabolites in faeces is commonly employed as a non-invasive method to monitor the activation of HPA axis and the stress response and well-being of wild and captive animals (Novak et al. 2013; Keay et al. 2006). Multiple factors can influence cortisol levels, including age, sex, social-rank and reproductive season (Rangel-Negrín et al. 2009; Novak et al. 2013; Takeshita et al. 2014), and one of the limitations of this study was that it was not possible to consider the individual physiological response. Whereas enrichment can lead to positive behavioural changes, evidence of its efficiency on HPA activity is scarce (Novak et al. 2013). In our study, the mean FCM concentration increased slightly during enrichment sessions compared to the control sessions, perhaps because the novelty of enrichment can cause some stress to the animals (Buchanan-Smith 2010). However, because of the small sample size and the numerous factors that can influence HPA activity, hormonal results are difficult to interpret, and further research should be conducted to correlate behaviour, FCM and enrichment effects. In the literature, it was reported that even if the effect of enrichment on glucocorticoid levels is still uncertain, it seems to have anxiolytic effects, with a positive influence in the physiological and behavioural response of the animals to stressors (Fox et al. 2006).

One of the limitations of this study was that the animals had previously received some enrichment, but not daily, and they had access to permanent, structural enrichment in their exhibit. This could have concealed the real effectiveness of the enrichment offered during the research sessions. However, there was previously no studied and programmed enrichment schedule in the centre, the animals did not receive enrichment every day, as during FE, ME and MIX sessions, and the enrichment used in the sessions of the study was totally new to them. Therefore, the positive changes observed in the study underline how a well-studied enrichment programme can have a positive effect on animal behaviour.

## Conclusions

This study underlines the importance of the respect for species-specific characteristics of the animals and of enrichment in rehabilitation programmes of ex-laboratory long-tailed macaques. The enrichment programmes should consider different factors, such as animals’ motivation and novelties. Feeding devices and manipulative enrichment seem to be more efficient than feeding only, because they avoid habituation and exploit manipulative motivation, typical of primates. Monitoring behaviour and FCM can be considered non-invasive methods useful for the assessment of stress and welfare in captive animals. Further research is needed to better understand the effect of enrichment on captive animals’ physiological and psychological well-being.
